# Integrated Care in an Era of Continual Reform: England’s ICS Experience; A Response to Recent Commentary

**DOI:** 10.34172/ijhpm.10005

**Published:** 2026-06-02

**Authors:** Mirza Lalani, Michele Peters

**Affiliations:** ^1^Department of Health Services Research and Policy, London School of Hygiene and Tropical Medicine, London, UK.; ^2^Nuffield Department of Population Health, University of Oxford, Oxford, UK.

## Introduction

 The comment by Baalla and Jellouli^1^ reflects on how Morocco might learn from integrated care systems (ICSs) in England, which were formally established in 2022. Their analysis offers a timely exploration of how principles of collaboration, governance, and population health management could inform health and care system reform beyond the English context. However, since the introduction of ICSs, the health and care landscape in England has undergone rapid and significant change, raising important questions about what lessons can be learned and transferred from implementing a top-down integrated care model.

## A Changing Governance and Policy Context in England

 A change in government in the UK has precipitated fiscal prudence and a focus on greater National Health Service (NHS) productivity. This has resulted in major reforms both centrally and at ICS level including changes to local authority structures with a shift toward unitary authorities covering populations of around 500 000. These changes have influenced ICS structures, encouraging co-terminosity as the geographical boundaries between the two entities align – increasing the possibility of ICS mergers.^2^ Coupled with an ambitious 10 Year Health Plan (10YHP)^3^ that focuses on three major shifts; hospital to community, analogue to digital and illness prevention, the structure of ICSs as intended in 2022 may be quite different once all reforms are complete. The rapidity of change means that there may be less time and capacity available for teams and staff to meet performance targets, let alone to enable service improvement.

 One of the more substantial changes instituted by the new Secretary of State for Health and Social Care was the decision to abolish NHS England (NHSE) which played a central role in ICSs as the primary funding body that set operational priorities and provided oversight (through its regional teams).^4^ It is not clear at this stage, how and to what extent ICSs will continue to receive strategic central support although they will now be directly accountable to the Department of Health and Social Care. What’s more, the decision to abolish Healthwatch^5^ (whose primary function was to ensure experiences of local users of health and social care services was heard at local and ICS levels) means the original ambition of ICSs to increase co-production and community engagement in developing and (re)designing health and social care services locally could stall.

 Recent history from England suggests reform to central healthcare governance coupled with large-scale redundancies can be costly and can precipitate the creation of new agencies that add further bureaucracy to an already highly bureaucratised system. Indeed, the healthcare reforms under the UK Coalition Government of the 2010s resulted in an estimated £1.4bn spent on redundancies, not to mention the loss of experienced staff. Moreover, critics suggested such changes had little or no impact on waiting lists or the quality of care.^6^

## Organisational Retrenchment and Capacity at ICS Level

 The abolition of NHSE was accompanied by ICS’s being asked to reduce costs and workforce by 50% resulting in a total loss of around 20 000 staff in primarily managerial and administrative roles across the different levels of the health care system.^7^ Moreover, economies of scale and the aforementioned reforms to local government mean that there are active discussions about ICS mergers and/or “clustering” arrangements (meaning two or more Integrated Care Boards working together). This could diminish the capability of ICSs to engage with local communities. In England, previous healthcare reforms enacted to enable efficiency savings resulted in manager roles being disbanded with politicians positing that savings can be redirected to the frontline.^8^ However, the loss of managers means frontline clinical staff spend more time undertaking administrative tasks which compromises their capacity and capability to provide high-quality and timely care.

 Another key ambition of the current Government is to institute neighbourhood health^9^ which focusses on integration of health and care services, prevention, personalised care, care closer to home, care led by communities and place-based care (centred on specified geographical locations that map to local authority boundaries). Indeed, neighbourhood health is central to achieving the goals of the 10YHP, yet with fewer staff at senior levels in ICSs it is not clear how this ambition can be achieved.

## What Progress Have England’s Integrated Care Systems Made?

 The progress ICSs are making in achieving their initial goals is generally uneven across the country. However, there are some emerging examples of promising local level collaboration between health, social and voluntary care organisations. The North Central London ICS multi-agency care and coordination team, led by a local General Practitioner Federation and Community Health Trust brings together general practitioners, social workers, therapists, mental health practitioners, and voluntary, community and social enterprise sector staff to provide integrated care to higher-risk adults living with frailty and long-term conditions. It is estimated that in 2024-2025 the team reduced accident and emergency and hospital admissions for their cohort of 2500 patients by 30%.^10^

 As highlighted by Baalla and Jellouli, integrated information systems are crucial to enabling more joined up, timely and higher quality of care for patients as they transition across health and care pathways. In 2025, all 42 ICSs in England rolled out operational Shared Care Records that contain key patient/user information including medications & allergies, diagnoses & conditions, test results, care plans, and discharge summaries.^11^ These records will enable the transparent visibility of key information about individual patients that can be accessed by health and social care partners.

## The Ongoing Absence of Social Care

 Even so, to maximise progress, ICSs will need to support improvements in social care which is hardly mentioned in the 10YHP. ICSs rely on health and social care partners (local authorities) to work together, yet the current Government has largely overlooked social care and opted to commission a review that will not report until 2027.^12^ A widely heralded reform to the social care funding model (recommended by Sir Andrew Dilnot) which had already previously been overlooked by successive Governments has been disregarded once again.^13^ Additionally, commitments to increase the skills and knowledge base of care workers through the implementation of training reforms enacted by the previous Government have stalled.^14^ Since 2022, social care has had formal representation in commissioning through Integrated Care Boards. However, there remains a longstanding perception within the sector that central government affords it lower priority and status than the NHS.^15^ Without further policy action, these concerns may persist.

## Funding Prevention

 One of the core objectives of ICSs is to improve population health, a priority that aligns to the NHS 10YHP’s emphasis on prevention and early intervention. ICSs provide the structural framework through which this preventive agenda can be embedded, shifting the system away from reactive treatment towards coordinated, place-based approaches that address the wider determinants of health. To enable this shift, more funding will need to be directed into public health. The current government ring-fenced Public Health Grant (which funds local prevention services such as smoking cessation, sexual health, weight management, etc) was increased to £3.8 billion for 2025/2026, a 5.4% uplift on the previous year. However, this follows a real terms reduction in local authority public health funding which fell by 26% in the preceding 10 years, resulting in cuts to public health services which has had a deleterious effect on health inequalities.^16^

## Conclusions

 Baalla and Jellouli draw attention to the potential value of England’s ICSs for health system reform beyond the UK. However, the English experience demonstrates that ICSs are a politically contingent model rather than a settled blueprint. Since their establishment, shifts in governance, funding, and system capacity have reshaped both the scope and feasibility of integration, prevention, and co-production. For Morocco, the key lesson may therefore lie less in replicating their institutional form, and more in recognising the foundational requirements for integrated care: stable governance, sustained investment, and sufficient organisational and workforce capacity.

## Disclosure of artificial intelligence (AI) use

 Not applicable.

## Ethical issues

 Not applicable.

## Conflicts of interest

 Authors declare that they have no conflicts of interest.

## Disclaimer

 The original research (Lalani et al^15^) was funded by the National Institute for Health Research (NIHR) Policy Research Programme, conducted through the Quality, Safety and Outcomes Policy Research Unit (PR-PRU-1217-20702). The views expressed in this response are those of the author(s) and not necessarily those of the NIHR nor the Department of Health and Social Care.
